# Pre-stroke apathy symptoms are associated with an increased risk of delirium in stroke patients

**DOI:** 10.1038/s41598-017-08087-7

**Published:** 2017-08-09

**Authors:** Elzbieta Klimiec, Katarzyna Kowalska, Paulina Pasinska, Aleksandra Klimkowicz-Mrowiec, Aleksandra Szyper, Joanna Pera, Agnieszka Slowik, Tomasz Dziedzic

**Affiliations:** Jagiellonin University Medical College, Department of Neurology, ul. Botaniczna 3, 31-503 Krakow, Poland

## Abstract

Neuropsychiatric symptoms can be interrelated to delirium. We aimed to investigate an association between pre-stroke neuropsychiatric symptoms and the risk of delirium in stroke patients. We included 606 patients (median age: 73, 53% female) with stroke or transient ischemic attack admitted within 48 hours from symptoms onset. We assessed delirium on a daily basis during the first 7 days of hospitalization. To make diagnosis of delirium we used DSM-5 criteria. We used Neuropsychiatric Inventory to assess neuropsychiatric symptoms occurring within 4 weeks prior to stroke. We diagnosed delirium in 28.2% of patients. On univariate analysis, higher score of pre-stroke depression (OR: 1.58, 95% CI: 1.04–2.40, P = 0.03), apathy (OR: 2.23, 95% CI: 1.44–3.45, P < 0.01), delusions (OR: 2.00, 95% CI: 1.09–3.68, P = 0.03), hallucinations (OR: 2.39, 95% CI: 1.19–4.81, P = 0.01) and disinhibition (OR: 2.10, 95% CI: 1.04–4.25, P = 0.04) was associated with the increased risk of delirium. On multivariate analysis adjusted for age, atrial fibrillation, diabetes mellitus, stroke severity, right hemisphere lesion, pre-stroke cognitive decline, pre-stroke disability and infections, higher apathy score (OR: 2.03, 95% CI: 1.17–3.50, P = 0.01), but no other neuropsychiatric symptoms, remained independent predictor of delirium. We conclude that pre-stroke apathy symptoms are associated with increased risk of delirium in stroke patients.

## Introduction

Delirium is a transient neurocognitive disorder characterized by cognitive, psychomotor and behavioral symptoms. The core features include attention and awareness disturbances, acute onset and fluctuations in symptoms severity^1^. Between 10% and 48% of stroke patients develop delirium^2^. Patients with post-stroke delirium have higher mortality, longer hospital stay and worse functional outcome^3, 4^.

Although delirium is widely recognized and has important clinical implications, its etiology is not fully explained. Delirium can be considered as a sign of the vulnerable brain with reduced resilience to insults^5^. According to this hypothesis, predisposing factors might be understood as preexisting conditions that diminish cognitive reserve and disturb brain compensatory mechanisms^6^. Therefore, identification of predisposing factors is important for understanding delirium’s pathophysiology.

Neuropsychiatric symptoms are not rare in elderly persons. In population-based study 27% of cognitively normal participants had at least one neuropsychiatric symptom^7^. These symptoms are even more frequent in patients with mild cognitive impairment^7^ or dementia^8^. Neuropsychiatric disturbances can be potentially inter-related to delirium. Both conditions can involve disruption of common neuronal networks responsible for mood and cognition, share risk factors and have similar pathophysiological mechanisms^9^. Numerous studies showed that depression is a risk factor for delirium^9, 10^. The relationship between other neuropsychiatric symptoms and delirium is poorly understood.

The aim of our study was to determine an association between preexisting neuropsychiatric symptoms and risk of delirium in stroke patients.

## Methods

PRospective Observational POLIsh Study on post-stroke delirium (PROPOLIS) is a prospective, single center study conducted in Department of Neurology, University Hospital, Krakow, Poland. The main goal of PROPOLIS is to determine frequency, predisposing factors and consequences of delirium in stroke patients^11^. We recruited participants to this study between May 2014 and March 2016. The Bioethics Committee of Jagiellonian University approved the study’s protocol. All methods were performed in accordance with approved guidelines and regulations. Each patient or his/her legal guardian gave an informed consent.

Inclusion criteria to this study were: (1) acute stroke (brain infarction, transient ischemic attack, intracerebral hemorrhage); (2) age ≥18 years; (3) admission within 48 h from symptoms onset; (4) Polish as a native language; (5) informed consent of a patient or a legal guardian. The exclusion criteria were: coma, brain tumor, alcohol withdrawal syndrome, cerebral venous thrombosis, subarachnoid hemorrhage, head trauma, vasculitis and diseases with life expectancy ≤1 year.

We screened patients for delirium on a daily basis during the first 7 days after admission to hospital. To assess core features of delirium we used Brief Confusion Assessment Method (bCAM) for verbal^12^ and Intensive Care Units version (CAM-ICU) for non-verbal patients^13^. When delirium was suspected, we also used Delirium Rating Scale-Revised-98^14^. In addition, nurses completed daily questionnaire about patients’ behavior and cognitive fluctuations. For the final diagnosis of delirium, we analyzed collected data in clinical context and assessed them according to DSM-5 criteria^15^. Patients with severe aphasia were assessed with Delirium Rating Scale-Revised–98, nurses daily questionnaire and DSM-5 criteria.

We gathered information about pre-stroke functional, cognitive and neuropsychiatric status using a structured interview with the close relative or caregiver who knew the patient well. This interview was completed during 48 hours from admission. We review patients’ medications for anticholinergic properties according to Anticholinergic Risk Scale (ARS)^16^. The scale ranks medication from 0 (no or low potential) to 3 (high potential). The total score is the sum of points for all patient’s medications.

We used Neuropsychiatric Inventory (NPI) to assess neuropsychiatric disturbances occurring within the four weeks prior to admission^17^. The NPI-Q10 subscale includes 10 behavioral items: delusions, hallucinations, agitation, depression, anxiety, euphoria, apathy, disinhibition, irritability and aberrant motor behavior. We calculated score for each item (from 0 to 12) as a product of severity scale (from 0 to 3) and frequency scale (from 0 to 4).

To assess pre-stroke functional status, we used modified Rankin Scale (mRS)^18^. We defined functional dependency as mRS 3–5.

To diagnose pre-stroke cognitive decline, we used a validated Polish version of the Informant Questionnaire on Cognitive Decline in the Elderly (IQCODE)^19^. The questionnaire consists of 26 items that rate the change in patients’ intellectual abilities over the past ten years. Each item is rated from 1 - much improved to 5 - much worse. Several cut-off scores for cognitive decline are used in practice^20^. For the purpose of our study, we chose the threshold of 3.3, because it has yielded the highest sensitivity^20^.

We assessed neurological deficit on admission using National Institute of Health Stroke Scale (NIHSS)^21^. Patients who received 2 points in speech item were classified as non-verbal. Patients who received 2 or more points in language item were classified as severely aphasic. For diagnosis of pneumonia and urinary tract infections we used Centers for Disease Control and Prevention criteria for clinically defined pneumonia and symptomatic urinary tract infection^22^.

We used χ^2^ test to compare proportions and Mann-Whitney test to compare continuous variables. We used uni- and multivariate logistic regression analyses to determine an association between independent variables and delirium. To multivariate analysis we included all variables with P value below 0.05 on univariate analysis. In analyses, we compared upper quartile of NPI scores to other quartiles. We performed 1:2 propensity score matching without replacement using nearest neighbor algorithm. For calculations, we used Statistica for Windows (version 10; Statsoft, Poland).

The datasets generated during and/or analysed during the current study are available from the corresponding author on reasonable request.

## Results

PROPOLIS study included 750 stroke patients (median age: 73, interquartiles: 63–82; 53.1% women; median NIHSS score on admission: 6, interquartiles: 3–15).

Information about pre-stroke neuropsychiatric symptoms was available for 606 patients (80.8%) and we included them into further analyses. Median age in this group was 73 (interquartiles: 63–82) and median NIHSS score was 6 (interquartiles: 3–14). Fifty-three per cent of patients were women. Baseline characteristic (age, sex and NIHSS score on admission) of patients included into analyses did not differ from the total cohort of patients participated in PROPOLIS.

We diagnosed delirium in 171 patients (28.2%). Table 1 shows baseline characteristics and Table 2 shows neuropsychiatric symptom scores in patients with delirium and patients without delirium.

**Table 1:** Baseline characteristic of patients with delirium and patients without delirium.

	Delirium (n = 171)	No delirium (n = 435)	p Value
Age (years), median (IQs)	78 (69–85)	70 (61–80)	**<0.01**
Female, n (%)	96 (56.1)	223 (51.3)	0.28
Hypertension, n (%)	125 (73.1)	302 (69.4)	0.37
Diabetes, n (%)	61 (35.7)	103 (23.7)	**<0.01**
Atrial fibrillation, n (%)	64 (37.4)	80 (18.4)	**<0.01**
Myocardial infarction, n (%)	30 (17.5)	57(13.1)	0.16
Pre-stroke cognitive decline, n (%)	61 (35.7)	68 (15.6)	**<0.01**
Pre-stroke functional dependency, n (%)	43 (25.1)	38 (8.7)	**<0.01**
Stroke type			0.11
Ischemic, n (%)	146 (85.4)	386 (88.7)	
Hemorrhagic, n (%)	18 (10.5)	25 (5.7)	
Transient ischemic attack, n (%)	7 (4.1)	24 (5.5)	
NIHSS score on admission, median (IQs)	14 (8–18)	5 (2–10)	**<0.01**
Lesion location			**<0.01**
Right hemisphere, n (%)	84 (49.1)	161 (37.0)	
Left hemisphere, n (%)	74 (43.3)	207 (47.6)	
Posterior fossa, n (%)	8 (4.7)	60 (13.8)	
>1 location	5 (2.9)	7 (1.6)	
Infections			
Pneumonia, n (%)	55 (32.2)	43 (9.9)	**<0.01**
Urinary tract infection, n (%)	79 (46.2)	124 (28.5)	**<0.01**
Pre-stroke diagnosis of neurological and psychiatric disorders			
Previous stroke	38 (22.2)	81(18.6)	0.16
Parkinson’s disease and parkinsonism, n (%)	2 (1.2)	6 (1.4)	0.84
Dementia, n (%)	10 (5.8)	10 (2.3)	0.03
Depression, n (%)	4 (2.3)	22 (5.1)	0.14
Anxiety disorders, n (%)	2 (1.2)	5 (1.1)	0.98
Schizophrenia, n (%)	1 (0.6)	0 (0)	0.11

IQs – interquartiles; NIHSS – National Institute of Health Stroke Scale.

**Table 2:** Neuropsychiatric symptom score in patients with delirium and patients without delirium.

Neuropsychiatric symptom score, median (IQs)	Delirium (n = 171)	No delirium (n = 435)	p Value
Neuropsychiatric Inventory subscale – Q10	2 (0–8)	0 (0–4)	**<0.01**
Delusions	0 (0–0)	0 (0–0)	0.29
Hallucinations	0 (0–0)	0 (0–0)	0.32
Agitation	0 (0–0)	0 (0–0)	0.26
Depression	0 (0–1)	0 (0–0)	0.12
Anxiety	0 (0–0)	0 (0–0)	0.73
Euphoria	0 (0–0)	0 (0–0)	0.82
Apathy	0 (0–1)	0 (0–0)	**0.02**
Disinhibition	0 (0–0)	0 (0–0)	0.41
Irritability	0 (0–1)	0 (0–0)	0.28
Aberrant motor behavior	0 (0–0)	0 (0–0)	0.56

Compared to patients without delirium, delirious patients were older, more often suffered from atrial fibrillation, diabetes mellitus, pre-stroke cognitive decline and pre-stroke disability. They had more severe neurological deficit on admission and more often suffered from in-hospital pneumonia and urinary tract infections. Right hemisphere lesions were more frequent in delirious patients. Total NPI-Q10 score was higher in patients with delirium compared to those without delirium. Among neuropsychiatric symptoms, only apathy had significantly higher score in patients with delirium.

In logistic regression analysis, we compared the upper quartile of NPI scores to other quartiles Table 3.

**Table 3:** Uni- and multivariate logistic regression assessing an association between neuropsychiatric symptoms and delirium.

	Univariate analysis		Multivariate analysis*	
	OR (95% CI)	p Value	OR (95% CI)	p Value
Neuropsychiatric Inventory subscale – Q10	1.63 (1.08–2.45)	**0.02**	1.25 (0.74–2.12)	0.40
Delusions	2.00 (1.09–3.68)	**0.03**	0.98 (0.46–2.13)	0.97
Hallucinations	2.39 (1.19–4.81)	**0.01**	1.31 (0.54–3.18)	0.54
Agitation	1.60 (0.98–2.62)	0.06	1.45 (0.78–2.68)	0.24
Depression	1.58 (1.04–2.40)	**0.03**	1.06 (0.64–1.78)	0.81
Anxiety	0.87 (0.51–1.49)	0.62	0.68 (0.35–1.32)	0.26
Euphoria	1.54 (0.55–4.33)	0.41	1.50 (0.47–4.84)	0.49
Apathy	2.23 (1.44–3.45)	**<0.01**	2.03 (1.17–3.50)	**0.01**
Disinhibition	2.10 (1.04–4.25)	**0.04**	1.53 (0.64–3.64)	0.33
Irritability	1.11 (0.71–1.75)	0.65	1.03 (0.59–1.82)	0.91
Aberrant motor behavior	1.44 (0.77–2.68)	0.25	1.18 (0.55–2.53)	0.67

Upper quartile of each score was compared to other quartiles. *Adjusted for age, diabetes, atrial fibrillation, NIHSS score, right hemisphere lesion, pre-stroke cognitive decline, pre-stroke disability, pneumonia and urinary tract infection.

On univariate analysis, higher score of pre-stroke depression, apathy, delusions, hallucinations and disinhibition were associated with the increased risk of delirium.

In the next step, we separately analyzed an association between delirium and each neuropsychiatric symptom after adjusting for potential confounders (age, atrial fibrillation, diabetes mellitus, NIHSS score on admission, right hemisphere lesion, pre-stroke cognitive decline, pre-stroke disability, pneumonia and urinary tract infections). Higher apathy score was associated with the increased risk of delirium (OR: 2.03, 95% CI: 1.17–3.50, P = 0.01). The association between other neuropsychiatric symptoms and delirium was non-significant on multivariate logistic regression analysis.

Finally, we put into the statistical model all-above mentioned confounders and all neuropsychiatric symptoms that had P value < 0.05 on univariate analysis (depression, apathy, delusions, hallucinations, disinhibition). Higher apathy score remained independent predictor of delirium (OR: 2.22, 95% CI: 1.22–4.02, P = 0.01).

Other significant predictors of delirium in our study were: older age (OR: 1.02, 95% CI: 1.00–1.04, P = 0.04), diabetes mellitus (OR: 1.91, 95% CI: 1.21–3.02, P < 0.01), right hemisphere lesion (OR: 1.78, 95% CI: 1.15–2.75, P = 0.01), pneumonia (OR: 2.29, 95% CI: 1.34–3.94, P < 0.01), and higher NIHSS score (OR: 1.11, 95% CI: 1.07–1.14, P < 0.01).

Data about pre-stroke medications were available for 536 patients (Table S1 in supplementary file).

Exclusion of patients who took anti-depressants (N = 18), neuroleptics (N = 7) or benzodiazepines (N = 12) before stroke, did not substantially change the results of multivariate analysis.

In our study, ARS score above 0 (OR: 2.02, 95% CI: 0.79–5.14, P = 0.14) or ARS score above 1 (OR: 1.82, 95% CI: 0.51–6.58, P = 0.36) was not associated with delirium on univariate analysis.

Next, we checked if pre-stroke medications could confound our results. After adding diuretics, insulin and angiotensin converting enzyme inhibitors to the to the statistical model (containing age, NIHSS score, atrial fibrillation, pre-stroke cognitive decline, pre-stroke disability and infections), the odds ratio of delirium in patients with higher apathy score was 1.80 (95% CI: 1.00–3.24, P = 0.049). The variable “diabetes mellitus” was not put into the model due to its interaction with variable “insulin”.

To confirm the obtained results, we performed cohort study matching. Propensity scores of patients with delirium were matched with propensity scores of patients without delirium taking into account the most important confounders (age, NIHSS score, diabetes mellitus, atrial fibrillation, infections, pre-stroke cognitive decline). A logistic regression analysis performed on balanced groups showed that higher pre-stroke apathy score is associated with an increased risk of delirium (OR: 1.89, CI: 1.19–2.99, P < 0.01).

## Discussion

Among 10 neuropsychiatric symptoms only apathy was independently associated with an increased risk of post-stroke delirium. Apathy is generally defined as a loss of motivation^23^. It can be considered as a separate syndrome or as a symptom of other disorders e.g. depression. Almost twenty per cent of community-dwelling elderly persons who are free of dementia and depression have symptoms of apathy^24^. Apathy is associated with a higher risk of functional impairment, lower cognitive performance and dementia^25^.

The association between apathy and delirium has not receive much attention so far. Hölttä and colleagues examined a cohort of 425 patients hospitalized in geriatric wards and nursing homes^26^. Apathy was assessed by trained nurses or geriatricians without use of any validated neuropsychiatric questionnaire. Twenty-three per cent of patients suffered from apathy. Patients with apathy more often had delirium (37.8% vs 21.1%, P < 0.01). The authors did not report results of multivariate analysis.

Apathy and delirium could be related to each other in several ways. First, structural disruption of common neuronal networks can be seen in apathy and delirium. In both entities diffusion tensor imaging studies showed microstructure abnormalities in frontal lobe, corpus callosum, thalamus and limbic structures^27, 28^. These structures are important for regulation of emotions and maintenance of consciousness and attention^28, 29^. Further studies are needed to identify common neuronal substrate for delirium and apathy. Second, the same risk factors could be relevant for delirium and apathy. Neurodegenerative and vascular pathologies can predispose to both of them. Apathy is common in neurocognitive disorders such as mild cognitive impairment or Alzheimer’s disease and its incidence increases with dementia progression^25^. In elderly, apathy is also associated with vascular risk factors (systolic blood pressure, body mass index, C-reactive protein level) and cardiovascular diseases including stroke^24^. Cognitive decline and vascular risk factors are predictors of delirium^5, 30^. In our multivariate analysis, apathy score predicted delirium independently from pre-stroke cognitive decline and vascular risk factors. Third, we previously mentioned that delirium can be a sign of brain vulnerability. In this context, apathy may be an early marker of diminished cognitive reserve, which appears before cognitive decline becomes detectable with screening tools. Given relatively low specificity of IQCODE in detecting patients who would develop dementia, this theory needs further investigation with the use of more accurate test^31^. Finally, apathy can be associated with depression, which is a risk factor for delirium in certain group of patients^9, 22^. Adding depressive symptoms score to our model did not change the association between apathy and delirium.

We did not find the association between delirium and depressive symptoms. Although depression was found to predispose to delirium in many studies^9^, some authors did not confirm this relation^32, 33^. We proposed several explanations for a lack of association between depression and delirium in our study. First, we used NPI-Q10 item to diagnose depressive symptoms. This instrument is based on interview with informants. Many previous studies assessed depression with tools based on self-report inventory such as Geriatric Depression Scale – 15 or Beck Depression Inventory^9^. Therefore, depression in our group might have been underestimated. Second, predictive role of depression may not refer to the whole syndrome, but only to some specific symptoms such as dysphoric mood and feeling of hopelessness^34^. Furthermore, the association between depression and delirium may be seen only in certain populations. In the literature review performed by Nelson and colleagues the connection between delirium and depression was found only in postoperative patients^10^. Finally, apathetic patients might meet criteria for depression^35^. Not all authors differentiated depression from apathy as we did in our study. Consequently, they might have misdiagnosed apathy as depression.

Apart from depression, anxiety has been considered as a predisposing factor for delirium. Two studies yielded negative results about relationship between delirium and anxiety^33, 36^. Schneider and colleagues found that psychopathological symptoms predicted higher delirium severity but they did not analyze separate symptoms^37^. We found on univariate analysis that delusions, hallucinations, and disinhibition were related to increased risk of delirium. However, this association did not withstand adjustment for potential confounders including pre-stroke cognitive decline.

Advantages of our study include: comprehensive assessment of delirium on a daily basis, complex evaluation of neuropsychiatric symptoms and relatively large group of unselected stroke patients.

We also need to address some limitations. NPI is commonly used tool for assessment of neuropsychiatric symptoms but it does not acknowledge self-reported symptoms. Given low agreement between self and informant report^38^, future studies should include these two complementary sources. We did not have information about pre-stroke neuropsychiatric symptoms for 19% of participants. It could be a source of potential bias, however, the total cohort of patients participated in PROPOLIS study and those included into this analysis did not differ in baseline characteristic. We cannot exclude the possibility that we missed delirium in patients who had symptoms only before admission to hospital. We also realized that stroke sequelae such as aphasia, inattention or neglect might interfere with delirium assessment. To avoid a misdiagnosis of delirium, we daily repeated assessment using validated tools, which cover multiple delirium manifestations and we paid special attention to symptoms fluctuations. Based on previous studies we did not exclude stroke patients due to dysphasia or attention disturbances on admission^4, 39, 40^. Exclusion of patients with potentially interfering stroke sequelae would lead to an underestimation of delirium in a whole cohort of stroke patients. In single cases, even after meticulous examination, it might be difficult to differentiate whether disturbances in attention, awareness and cognition are due to delirium or they are secondary to neurological and neuropsychiatric consequences of stroke. In clinical practice, including such patients under the umbrella of delirium will result in increased patient safety through delirium identification and prevention. Furthermore, due to a lack of normative data, NPI does not allow us to differentiate between normal and pathological intensity of neuropsychiatric symptoms. Lastly, predisposing factors for delirium vary among different cohorts of patients^5^, therefore, our results cannot be generalized and need to be confirmed in non-stroke patients.

Apathy may be considered as a predisposing factor for post-stroke delirium but can be also a marker of other conditions which contribute to delirium risk, such as undetected cognitive decline. This needs clarification in future studies. Nevertheless, our findings could have potential clinical implications. Identification of patients with premorbid apathy symptoms might be important for a selection of persons who are at risk of delirium. These patients may demand extensive delirium prevention and closer monitoring for early detection and escalated treatment of delirium.

In conclusion, we found apathy symptoms as a predisposing factor for post-stroke delirium.

## Electronic supplementary material

Supplementary Information
