# Supplementary material for: Pre-stroke apathy symptoms are associated with an increased risk of delirium in stroke patients
Source: Sci Rep. 2017 Aug 9;7:7658. doi: 10.1038/s41598-017-08087-7 (PMC5550489; doi:10.1038/s41598-017-08087-7)
Supplement: Supplementary file 1 — Supplementary Information [file 41598_2017_8087_MOESM1_ESM.pdf]

Pre-stroke apathy symptoms are associated with an increased risk of delirium in stroke patients.

Elzbieta Klimiec[1], MD; Katarzyna Kowalska[1], MSc; Paulina Pasinska[1], MD; Aleksandra Klimkowicz-Mrowiec[1] PhD, MD; Aleksandra Szyper[1], MSc; Joanna Pera[1], PhD, MD; Agnieszka Slowik[1], PhD, MD; Tomasz Dziedzic[1]*, MD, PhD.

1. Jagiellonin University Medical College, Department of Neurology, ul. Botaniczna 3, 31-503 Krakow, Poland
* dziedzic@cm-uj.krakow.pl
Phone +48 12 424-86-00

Supplementary Data

Table S1. Pre-stroke medications in patients with and without delirium.

	**Delirium (n=143)**	**No delirium (n=393)**	**p Value**
Acetylsalicylic acid, n (%)	47 (32.9)	128 (32.6)	0.93
Statins, n (%)	45 (31.5)	92 (23.4)	0.06
Oral hypoglycemic agents, n (%)	23 (16.1)	68 (17.3)	0.70
Insulin, n (%)	18 (12.6)	22 (5.6)	<0.01
Beta blockers, n (%)	65 (45.5)	138 (35.1)	0.03
Angiotensin converting enzyme inhibitors, n (%)	61 (42.7)	122 (31.0)	0.01
Diuretics, n (%)	47 (32.9)	82 (20.9)	<0.01
Antidepressants, n (%)	3 (2.1)	15 (3.8)	0.27
Neuroleptics, n (%)	2 (1.4)	5 (1.3)	0.98
Benzodiazepines, n (%)	4 (2.8)	8 (2.0)	0.69
